# Is there an indication left for gastric band? A single center experience on 178 patients with a follow-up of 10 years

**DOI:** 10.1007/s13304-020-00858-8

**Published:** 2020-07-31

**Authors:** Antonio Vitiello, Giovanna Berardi, Nunzio Velotti, Giovanni Domenico De Palma, Mario Musella

**Affiliations:** 1 Advanced Biomedical Sciences Department, Naples “Federico II” University, AOU “Federico II”, Via S. Pansini 5, 80131 Naples, Italy; 2 Department of Clinical Medicine and Surgery, University of Naples Federico II, Naples, Italy

**Keywords:** LAGB, Gastric band, Band removal, Long term results

## Abstract

**Background:**

Laparoscopic Adjustable Gastric Banding (LAGB) has been widely performed in the past at our university bariatric center. Aim of this study was to retrospectively assess long term outcomes of LAGB at our university hospital, with special regard to non-response (EWL < 25%) and rate of band removal.

**Methods:**

Retrospective search of prospectively maintained database of our university bariatric center was carried out to find all consecutive patients that had undergone LAGB at our department with a minimum follow-up of 10 years. Collected data were sex, age, body mass index (BMI), obesity related diseases remission, complications and weight loss.

**Results:**

After 10 years, patients with the band (*n* = 144) in place had a BMI of 35.2 ± 7.5 kg/m2, while %EWL and % TWL were 40.8 ± 52.4 and 18.9 ± 20.7. Seventy-four (41.6%) achieved a success (%EWL > 50), while 38 (21.3%) were non-responders (%EWL < 25), 32 (18%) had an insufficient weight loss (25 < %EWL < 50) and 34 (19.1%) underwent band removal. Among these, 6 (3.4%) were removed for complications and 28 (15.7%) for insufficient weight loss. Weight regain occurred in 38 out of 144 (26.4%) subjects with the band in place at 10 years. Only one case of early vomiting with readmission for medical treatment was recorded. Slippage, erosion/migration and port/tube complications occurred in 4 (2.2%), 2(1.1%) and 9(5%) cases respectively.

**Conclusion:**

LAGB is a safe and moderately effective bariatric procedure but it showed disappointing rates of removal, non-response and remission from comorbidities. However, LAGB could still be proposed for selected/motivated patients.

## Introduction

Laparoscopic approach for gastric banding not only represented a milestone for the history of this procedure but also promoted the endorsement and diffusion of bariatric surgery itself. Laparoscopic adjustable gastric banding (LAGB) became one of the most performed bariatric procedures accounting for 24.4% of all bariatric procedures worldwide in 2003 and 42.3% in 2008 [1, 2].

However, several studies published in the subsequent years demonstrated a long-term failure rate ranging from 40 to 70% [3–6]. Indeed, in 2011 percentage of LAGBs performed worldwide decreased to 17.8%, mainly because of the success of the laparoscopic sleeve gastrectomy (LSG) [7]. In the last years, the decline of LAGB has continued and, according to last IFSO reports (International Federation for Surgery for Obesity) [8, 9], it has been almost abandoned.

More than 10 years ago, LAGB was the most performed procedure at our Center for the Interdisciplinary Treatment of Obesity, accounting for 50% of all bariatric surgeries, but currently LSG and OAGB/MGB are the preferred surgical choices. Currently, about two hundred interventions are performed each year in our Institution and LAGB represents only 5–10%.

Aim of this study was to retrospectively assess long term outcomes of LAGB at our university hospital, with special regard to non-response (EWL < 25%) and rate of band removal.

## Materials and methods

Retrospective search of prospectively maintained database of our university bariatric center was carried out to find all consecutive patients that had undergone LAGB at our department with a minimum follow-up of 10 years. Inclusion criteria were age between 18 and 60 years, BMI > 40 kg/m^2^ or > 35 with a related disease. Subjects with previous history of bariatric or abdominal surgery were excluded.

Collected data were sex, age, body mass index (BMI), obesity related diseases remission, complications and weight loss.

### Surgical technique

All patients were positioned on the operating table in a 15–30 degrees reverse Trendelenburg position; the surgeon stood between the patient legs; the cameraman was on the left. Closed pneumoperitoneum of 12–14 mm Hg was achieved using a Veress needle. A total number of 4 trocars were placed as follows: one 10 mm trocar above the umbilicus for the 30 degrees laparoscope; another 10 mm trocar on the midclavicular line 5–6 cm below the costal margin; one 5 mm trocar was placed below the xiphoid appendices for liver retraction and a 5 mm trocar was placed between the right anterior axillary and the midclavicular line, 4–5 cm subcostally. The operation then started with the dissection of the gastrophrenic ligament and with the opening of the pars flaccida of the small omentum. A grasper was moved along the right crus to create a retrogastric tunnel; a band-placer was then inserted in this path to appear on the greater curvature of the stomach at the site of the prior dissection of His angle. The band was drawn along this tunnel and then closed. The procedure ended with 2 gastro-gastric sero-serous nonabsorbable sutures passed between the gastric fundus and the gastric pouch above the band. Leak test was not performed routinely, and nasogastric tube was not placed [10].

### Preoperative evaluation and follow-up

All patients were preoperatively evaluated by an interdisciplinary team consisting of endocrinologists, psychiatrists, dieticians and surgeons. Liquid diet was started on postoperative day 1 and discharge was planned the day after for uneventful procedures. Pureed foods were allowed after postoperative day 15 and normal diet after 30 days. Follow-up appointments were routinely planned at 1, 3, 6 and 12 months. After the first year, visits were planned every 6 months. Band regulations were decided on the base of patient’s symptoms and weight. Unplanned appointments and regulations were scheduled in case of acute vomiting or dysphagia and a barium swallow was required.

### Weight loss

Weight loss was calculated as percentage of excess weight loss (%EWL) at 1, 5 and 10 years.

Success at 10 years at was defined as %EWL ≥ 50, non-response was set as %EWL < 25 [11], while 25 < %EWL < 50 was set as insufficient weight loss (IWL). Weight regain at 10 years was defined as %EWL < 50 for a patient who had previously achieved %EWL > 50.

In addition, total weight loss percent (%TWL) was calculated. Multiple linear regression using preoperative characteristics (sex, age, BMI and comorbidities) as independent variables and %TWL at 10 years as dependent variable was performed.

### Remission from obesity related disease

Remission of type 2 diabetes (T2DM) was considered as a value of glycated haemoglobin A1c (HbA1C) < 6.5% off antidiabetic medications [12]. Hypertension (HTN) remission was defined as blood pressure < 140/90 with off antihypertensive medication [13]. Dyslipidaemia cut-offs points were chosen according to the American Heart Association criteria to identify metabolic syndrome [14].

### Complications

Early postoperative (< 30 days) complications (bleeding, perforation, untreatable vomiting) and late complications (slippage, erosion/migration and port/tube infection) were recorded.

### Statistical analysis

Statistical analysis was performed using Statistical Package for Social Sciences (SPSS) software for windows. Continuous data are expressed as means ± standard deviation. Significance was set at a *p* value of < 0.05.

## Results

A total number of 225 patients were eligible for this study, but 47 (21%) subjects were loss during follow up; therefore 178 (79%; 52 males/126 females) patients were included. Initial age and BMI were 38 ± 11.5 years and 44.4 ± 6.5 kg/m^2^ respectively.

### Weight loss

After 10 years, patients with the band in place (*n* = 144) had a mean BMI of 35.2 ± 7.5 kg/m^2^, while %EWL and % TWL were 40.8 ± 52.4 and 18.9 ± 20.7. Seventy-four (41.6%) achieved a success (%EWL > 50), while 38 (21.3%) were non-responders (%EWL < 25), 32 (18%) had an insufficient weight loss (25 < %EWL < 50) and 34 (19.1%) underwent band removal.

**Fig. 1:**
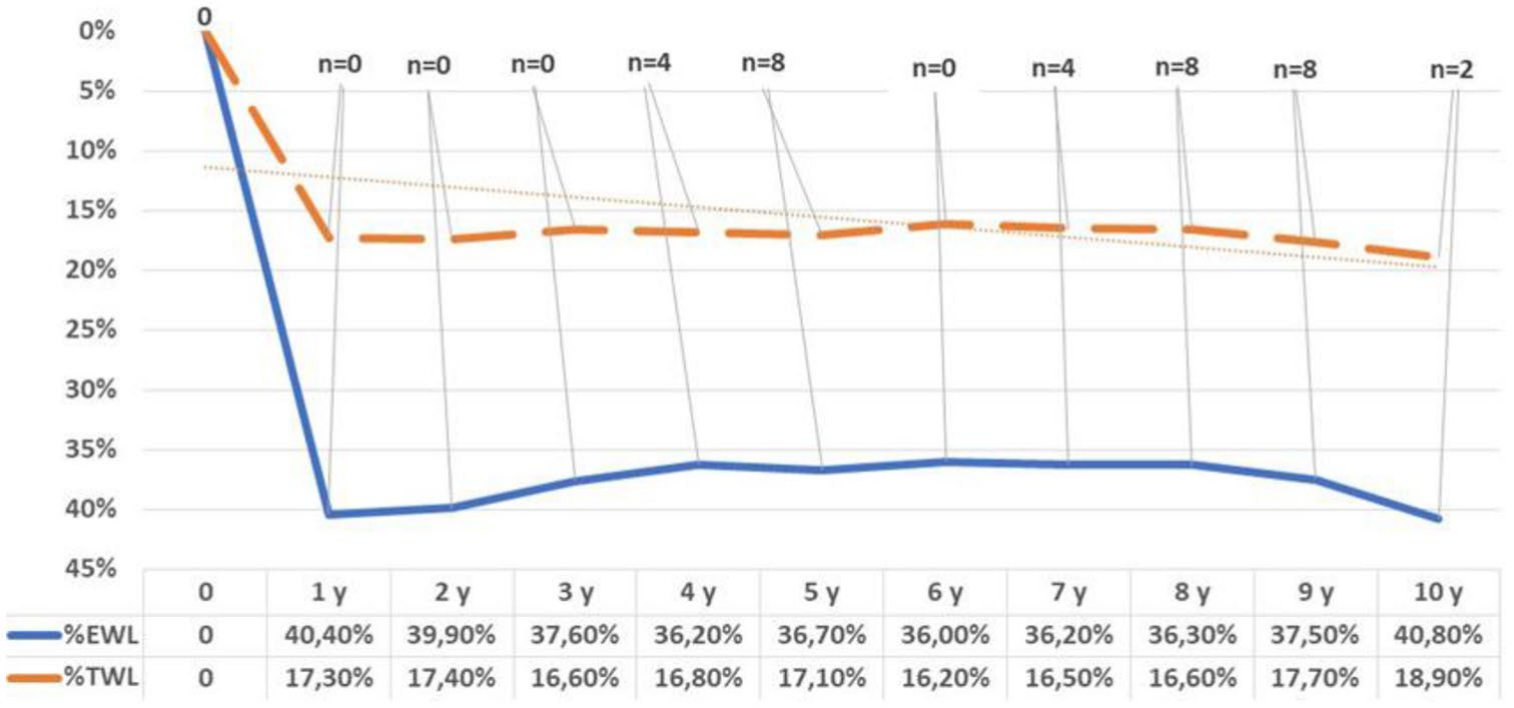
Trend of percentage of excess weight loss (%EWL) and Total Weight Loss percent (%TWL) during follow-up. *n* = number of removals

Weight regain occurred in 38 out of 144 (26.4%) subjects with the band in place at 10 years. Multiple regression analysis demonstrated a significant relationship in favor of male gender (*p* = 0.01) and BMI (*p* < 0.01) with %TWL at 10 years. %EWL, %TWL and number of band removals during follow-up are pictured in Fig. 1.

### Remission from obesity related disease

At baseline 35 (19.7%) patients were diagnosed with Hypertension, 20 (11.2%) were diabetic and 34 (19.1%) had dyslipidaemia. After 10 years, remission rate from HTN, T2DM and Dyslipidaemia were 20% (*n* = 7), 0% (*n* = 0) and 23.5% (*n* = 8) respectively.

### Complications

One patient was readmitted on postoperative day 4 for severe vomiting and discharged after medical treatment; no other early complication was found in our cohort. Slippage, erosion/migration and port/tube complications occurred in 4 (2.2%), 2(1.1%) and 9(5%) cases respectively. Port/tube problems were resolved with minor interventions in local anaesthesia.

Band removal rate at 10 years was 19.1% (*n* = 34), 6 (3.4%) were removed for complications and 28 (15.7%) for insufficient weight loss. Among these, 24 subjects refused further treatment while 10 were converted to other procedures. Six patients were converted to LSG, 2 to Roux-en-Y gastric bypass (RYGB) and 2 to Mini-bypass/One anastomosis gastric bypass (MGB/OAGB). All conversions were performed in one step.

## Discussion

LAGB has always been considered as a definitive procedure due to its adjustable nature and high-tolerated material. Silicone is, indeed, the best-tolerated material, and recent bands are also designed to create few gastric adhesions [15]. However, the gastric band remains a foreign body and with time it may migrate or slip. Also, the port-tube system, which is placed in the subcutaneous tissue, could get infected and migrate due to multiple traumas of regulations. However, infection, migration and slippage in large series are reported in less than 5% of cases [16–18], being unsatisfactory weight loss the major reason for removal or conversion to another procedure [18, 19]. Interestingly, several studies have demonstrated effectiveness and safety of conversion of LAGB to other bariatric procedures in one- or two-steps [20–22].

Main reason of the decline of LAGB is the unsatisfactory weight loss due to non-response or weight regain, as reported in series with medium [23] or long term [24–26] follow-up. Nevertheless, a study with 18 years of follow-up reported that despite band removal may occur in half of the patients over time, considering its reversibility and safety, LAGB still has a place in the treatment of morbid obesity [27].

A recent systematic review for studies with 10-year follow-up data, including a total of 9706 patients [28] showed a mean of 49% EWL at 10 years with a 30% band removal rate. However, reported failure and removal rates for LAGB reached 70% in studies with a follow-up longer than 20 years [29–32].

These data are consistent with our findings; 60% of our patients underwent band removal or did not achieve %EWL > 50. However, 40% of subjects with the band in place still had a success after 10 years and this outcome could demonstrate that LAGB may be effective for selected/motivated patients in medium and in long term. Conversely to previous evidences [33–35], LAGB proved to be more effective in male patients and in those with higher BMI. Moreover, weight regain (WR) occurred in 26% of cases, which is comparable to WR after other bariatric interventions such as LSG [36] or RYGB [37, 38]. Removal rate was slightly less than 20% in our cohort and a systematic review [39] has shown that all procedures have a substantial need for re-operative surgery and the levels of reoperation for LAGB are within the range of other bariatric interventions. This is even more important considering that perioperative complications in high-volume centers for LAGB placement, removal or conversion are 1–2% [19].

These findings could lead to the consideration that LAGB could be considered as a first step for patients with high BMI; LAGB would be safer than LSG in terms of complications and conversion to RYGB [40] or MGB/OAGB [41, 42] would represent a totally reversible two-step procedure.

In summary, since LAGB is not a novel procedure, body of literature regarding this intervention is consistent, but, in our opinion, it has been misinterpreted. LAGB has been almost abandoned just because it is considered not effective or obsolete. As demonstrated in our series, LAGB is effective in long term in selected patients and more efforts should be made to find predictive selection criteria. These criteria could allow surgeons to keep the gastric banding in their armamentarium since it is safe, totally reversible and convertible to other bariatric interventions. Moreover, rate of reoperation after LAGB is in line with the percentage of reintervention after bariatric surgery; vast majority of reoperations after LAGB are removals, port-tube interventions and conversions, which are usually performed with a low rate of complications. Moreover, several factors imply in the genesis and development of obesity and that can modify the postoperative course of bariatric patients and weight loss regardless of the surgical procedure [43].

In regard of resolution of obesity related diseases, we must disclose that LAGB has never been the favourite choice for diabetic patients or subjects with metabolic syndrome in our center. Subsequently, the initial percentage of patients suffering with T2DM, HTN and dyslipidaemia was not particularly high in our cohort. Since LAGB is a purely restrictive procedure, it was not unexpected a low rate of resolution, especially in long term. Unlike other procedures [44], improvement of comorbidities after LAGB can only be related to weight loss and not to metabolic changes, indeed a 0% remission from T2DM was recorded in our study.

## Conclusion

LAGB is a safe and moderately effective bariatric procedure in long term but it showed disappointing rates of removal, non-response and remission from obesity related diseases. However, LAGB could still be proposed for selected/motivated patients.
